# Synthesis, antileishmanial, antimalarial evaluation and molecular docking study of some hydrazine-coupled pyrazole derivatives

**DOI:** 10.1186/s13065-023-01111-0

**Published:** 2024-01-08

**Authors:** Halefom Gebreselasse Berhe, Yihenew Simegniew Birhan, Botros Youssef Beshay, Huda Jawad Habib, Ariaya Hymete, Adnan Ahmed Bekhit

**Affiliations:** 1 School of Pharmacy, College of Health Science, Mekelle University, Mekelle, Ethiopia; 2 Department of Chemistry, College of Natural and Computational Sciences, Debre Markos University, P.O. Box 269, Debre Markos, Ethiopia; 3 Department of Pharmaceutical Chemistry, College of Pharmacy, Arab Academy for Science, Technology and Maritime Transport, Alexandria, 21913 Egypt; 4 Pharmacy Program, Allied Health Department, College of Health and Sport Sciences, University of Bahrain, Manama, Kingdom of Bahrain; 5 Department of Pharmaceutical Chemistry and Pharmacognosy, School of Pharmacy, Addis Ababa University, P. O. Box 1176, Addis Ababa, Ethiopia; 6 Department of Pharmaceutical Chemistry, Faculty of Pharmacy, Alexandria University, Alexandria, 21215 Egypt

**Keywords:** Hydrazine-coupled pyrazoles, Antileishmanial activities, Antimalarial activities, Molecular docking

## Abstract

Pyrazole-bearing compounds are known for their diverse pharmacological effects including potent antileishmanial and antimalarial activities. Herein, some hydrazine-coupled pyrazoles were successfully synthesized and their structures were verified by employing elemental microanalysis, FTIR, and ^1^H NMR techniques. The in vitro antileishmanial and in vivo antimalarial activities of the synthesized pyrazole derivatives (**9–15**) were evaluated against *Leishmania aethiopica* clinical isolate and *Plasmodium berghei* infected mice, respectively. The result revealed that compound **13** displayed superior antipromastigote activity (IC_50_ = 0.018) that was 174- and 2.6-fold more active than the standard drugs miltefosine (IC_50_ = 3.130) and amphotericin B deoxycholate (IC_50_ = 0.047). The molecular docking study conducted on Lm-PTR1, complexed with Trimethoprim was acquired from the Protein Data Bank (PDB ID:2bfm), justified the better antileishmanial activity of compound **13**. Furthermore, the target compounds **14** and **15** elicited better inhibition effects against *Plasmodium berghei* with 70.2% and 90.4% suppression, respectively. In conclusion, the hydrazine-coupled pyrazole derivatives may be considered potential pharmacophores for the preparation of safe and effective antileishmanial and antimalarial agents.

**Supplementary Information:**

The online version contains supplementary material available at 10.1186/s13065-023-01111-0.

## Introduction

Leishmaniasis and malaria are communicable, devastating, and neglected tropical diseases (NTDs) affecting more than 500 million people worldwide. The causative agents of leishmaniasis (*Leishmania* strains) and malaria (*Plasmodium* strains) can be transmitted through the bite of sandflies and mosquitoes, respectively [1, 2]. Globally, nearly 350 million people are at risk of leishmaniasis. The overall prevalence of leishmaniasis in the world is 12 million [3]. For instance, visceral leishmaniasis (VL) was implicated in approximately 6% (1.4 million) of all disability-adjusted life-years (DALYs) caused by NTDs in 2015 [2], echoing the economic implication of leishmaniasis in low- and middle-income countries. In addition, nearly half of the global population is at risk of contracting malaria infection by the WHO in 2021. Moreover, 241 million cases and 627,000 deaths were occurred in 2020 slightly higher compared to 2019 [4]. Despite the global healthcare importance of leishmaniasis and malaria, there are only a few drugs often deployed to treat them in the clinical setting. Ironically, multiple reports revealed that the efficacy of the existing antileishmanial and antimalarial drugs is often conceded due to suboptimal treatment outcomes and the advent of drug-resistant *Plasmodium falciparum* [5–11] which is responsible for most of the mortality and morbidity associated with malaria [12–14]. Thus, the discovery of new antileishmanial and antimalarial agents with desirable therapeutic efficacy and tolerable side effects is highly demanded to treat infections caused by leishmaniasis and malaria [15–17].

**Fig. 1:**
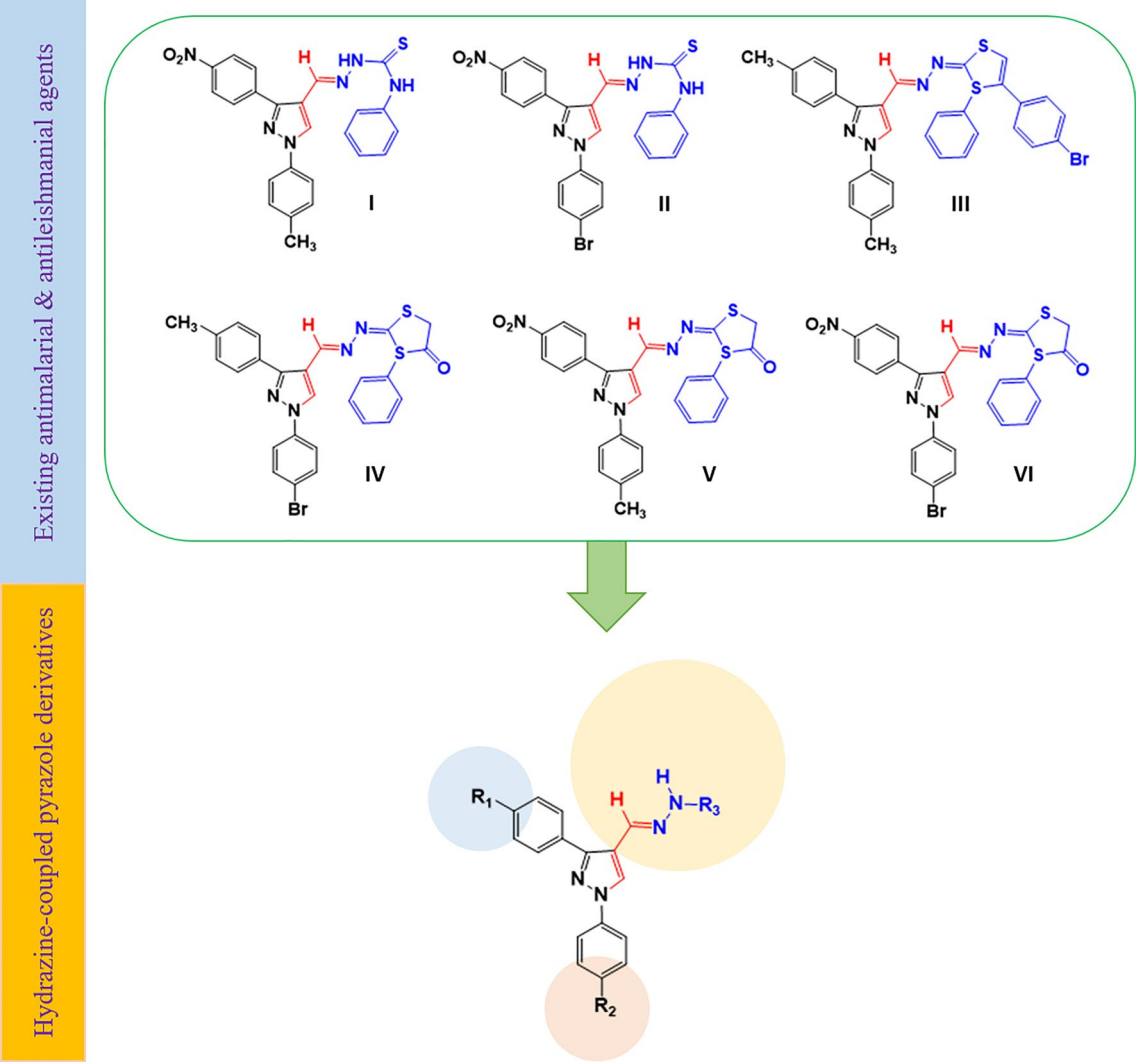
Design rationale for hydrazine-coupled pyrazole derivatives based on the existing dual-acting antimalarial and antileishmanial agents

Pyrazoles are a class of bioactive compounds with diverse pharmacological effects such as anticancer [18, 19], antiviral [20, 21], antifungal [22], antibacterial [23], anti-inflammatory [24], antioxidant activities [25]. Moreover, hydrazine-coupled pyrazole derivatives are shown to have promising antimalarial and antileishmanial activities [26–28]. In this regard, Bekhit and coworkers [29] successfully synthesized different hydrazine-coupled pyrazole derivatives (**I-VI**) with potent antimalarial activities having high percent suppression (94.19 to 97.67%) against mice infected with *Plasmodium berghei* at a dose of 48.4 µM/kg per day. In addition, the compounds (Fig. 1) displayed desirable IC_50_ values (0.0364 to 0.0418 µM) against chloroquine-resistant RKL9 strains compared to chloroquine phosphate (IC_50_ = 0.1920 µM). Furthermore, they also exhibited superior antileishmanial activities with IC_50_ ranging from 0.0241 to 0.0341 µg/mL which was far better than the conventional drugs miltefosine (IC_50_ = 3.1921 µg/mL) and amphotericin B deoxycholate (IC_50_ = 0.0472 µg/mL). Interestingly, all the synthesized pyrazoles were safe and well tolerated by mice when treated with 300 mg/kg and 100 mg/kg through oral and parenterally administration, respectively. Inspired by the appealing pharmacological profiles of the aforementioned compounds, this study aimed to synthesize some hydrazine-coupled pyrazole derivatives by incorporating different moieties with potential effects on the solubility and interactions of target compounds with biological macromolecules. In addition, the antimalarial activities, antileishmanial activities and acute toxicities of the target compounds were assessed.

## Results and discussion

### Chemistry

The target hydrazine-coupled pyrazole derivatives (**9–15**) were synthesized by employing nucleophilic addition–elimination reaction of intermediates (**5–8**) with different hydrazine derivatives (salicyl hydrazide, hydrazine hydrate, and phenyl hydrazine). In this study, the target compounds were prepared with good yields ranging from 61.64 to 95.5%. The percent yield of the synthesized compounds was comparable with previously synthesized hydrazine-coupled pyrazole derivatives [27]. The structures of the synthesized compounds were verified by employing different techniques such as elemental microanalysis, FTIR, and ^1^H NMR. For instance, the FTIR spectrum of compound **13** resonated the presence of a characteristic absorption band at 3292 cm^−1^ attributed to the NH group. It appeared at a relatively higher frequency due to the absence of amidic carbonyl moiety. Two medium bands corresponding to C = N asymmetric and symmetric stretching vibrations appeared at 1615 and 1593 cm^−1^, respectively. The presence of a band for the NH group and the absence of bands for C = O stretching vibrations around 1678 cm^−1^ verified the formation of compound **13**. Moreover, the ^1^H NMR spectrum also reiterated the successful synthesis of the target compounds. In this regard, the peaks between δ 7.89–9.97 integrated for three protons were attributed to the overlapped doublet peaks of benzenesulfonamide-C_3,5_ and singlet peak of pyrazole-C_5_ hydrogens, respectively. Moreover, the presence of a single peak for the N = CH at δ 8.99, a singlet peak for NH at δ 10.25, and the absence of the singlet peak for the CHO group at δ 10.1 proved the synthesis of compound **13** beyond the reasonable doubt. The chemical structures of the synthesized compounds were verified using spectral and physical measurements. The elemental microanalysis, specific stretching and bending IR vibration frequencies, and the ^1^H NMR chemical shift data for each of the hydrazine-coupled pyrazoles (**9–15**) are presented in the experimental section. Overall, the protocols followed in the preparation of target compounds resulted in the formation of colored well-defined crystals characteristic of hydrazine-coupled pyrazole derivatives.

### Biological activity results

#### In vitro antileishmanial activity results

The viability of promastigotes can be estimated by microscopically counting live cells, measuring enzyme activities via the 3-(4,5-dimethyl-thiazol-2-yl)-2,5-diphenyl-tetrazolium bromide (MTT) assay, etc. The frequent use of these methods is hampered due to the lengthy and time-consuming procedures followed in the test. AlamarBlue^®^ has been widely employed in vitro antileishmanial sensitivity assays as it is nontoxic for cells even during long incubation times, water-soluble, and highly stable in complete media. In the living cells, the non-fluorescent resazurin is metabolically reduced to a fluorescent active resorufin thereby changing its color from blue to red. The extent of fluorescence produced by each sample is strongly correlated with the number of living cells. In this study, the antipromastigote assays of the target compounds were carried out in triplicates and the results were presented as IC_50_ values. The result disclosed that all target compounds except compounds **11** (IC_50_ = 5.738 µg/mL) and **14** (IC_50_ = 3.263 µg/mL) had far better antipromastigote activity (Table 1) than miltefosine (IC_50_ = 3.13 µg/mL). However, all the hydrazine-coupled pyrazoles except compound **13**, exhibited lower antipromastigote activity as compared to amphotericin B deoxycholate (IC_50_ = 0.047 µg/mL). Most of the test compounds lacking the C = O group adjacent to the C = N bond demonstrated superior antileishmanial activities. Exceptionally, compound **13** had the highest antipromastigote activity (IC_50_ = 0.018 µg/mL) which is about 174- and 2.6-fold more active than miltefosine and amphotericin B deoxycholate, respectively. This might be attributed to the strong H-bonding interactions between NH moiety and the residual amino acids in the active site of the enzyme. To validate this, compound **13** was fitted into the simulated active site of Lm-PTR1 and the result was consistent with the in vitro antileishmanial data. Moreover, compound **9** which contains bromine had the second-highest antipromastigote activity with an IC_50_ value of 0.084 µg/mL. On the contrary, compound **11** with a methyl substituent at the same position elicited far less antileishmanial activity than compound **9**, highlighting the importance of electron-withdrawing substituent at the 4-position of the benzene ring attached to N-1 of the pyrazole ring for improved antipromastigote activity. Overall, the hydrazine-coupled pyrazole derivatives with the aforementioned attributes may serve as a potential scaffold for the preparation of potent antileishmanial agents.

**Table 1:** Data for antipromastigote activity (IC_50_) testing of the synthesized compounds

Test compounds	Structure	IC_50_ Values (µg/mL)	IC50 Values (µM/mL)
9		0.084	0.256
10		0.207	0.450
11		5.738	10.413
12		0.111	0.280
13		0.018	0.033
14		3.263	6.706
15		0.181	0.441
Miltefosine	–	3.130	7.680
Amphotericin B	–	0.047	0.035

#### In vivo antimalarial activity results

The four-day suppressive test was implemented to screen the antimalarial activity of the target compounds as it is effective in evaluating early infections and convenient to assess the prodrug effects of test compounds [30]. In this study, the in vivo antimalarial activity of the target compounds was investigated by using mice infected with *Plasmodium berghei* (achieve steady-state infection) at a dose of 48.4 µmol/kg per day. The results of the antimalarial activity screening of target compounds against the control groups were expressed based on mean parasitemia and percent inhibition of parasitemia (Table 2) which is known as a reliable parameter for in vivo antimalarial screening tests [31]. The treated with compounds **13**, **14,** and **15** (with no amidic carbonyl group placed at the 4-position of the pyrazole ring) demonstrated percent suppressions greater than 50% but suboptimal as compared to the positive control (which did not experience any death in the study period). While compounds **9**, **10** and **11** containing amidic carbonyl group had less than 50% mean percent suppression compared to the positive control groups. Among the tested compounds, bis-[*N*,*N*-dimethylaminomethylene-4-(3-(4-methylphenyl)-4-(hydrazonomethylene)-1*H*-pyrazole-1-yl)benzenesulfonamide] (**15**) showed a desirable level of mean parasitemia (5.0 ± 1.4) and highest percent suppression (90.4%) against *Plasmodium berghei* infected mice but the mean survival time of the mice in the group was not appreciably different (7.7 ± 1.4) as compared to the negative control (7.0 ± 1.2). On the other hand, there was an increase in the mean survival time (9.8 ± 1.3) of mice treated with a half dose (24.2 µmol/mL) of compound **15**, highlighting the potential dose-dependent toxicity associated with bis-structure due to cleavage of acid labile C = N bond and subsequent increase in concentration in the systemic circulation. 4-[3-(4-methylphenyl)-4-hydrazonomethylene-1*H*-pyrazol-1-yl]benzenesulfonamide (**14**) displayed the next better antimalarial activity (70.2%) that is complemented by the relatively lower mean parasitemia level (15.5 ± 4.1) and longer mean survival time of 9.0 ± 2.0 days. This could be attributed to the presence of a primary amine group adjacent to the C = N bond that can increase the strength of binding interaction with the active site of the receptor by hydrogen bonding. Moreover, the hydrophobic interaction imparted by the methylated phenyl group with the amino acid residues in the enzyme pocket may be beneficial for the observed antimalarial activity of compound **14**. The compounds containing C = O bond (**9–12**) near C = N had relatively lower antimalarial activity due to the electron-withdrawing effect and subsequent decrease in hydrogen bonding interactions with the different functional groups reside in the enzyme pocket. In general, some hydrazine-coupled pyrazoles which possessed substituents or moieties that favor a desirable binding interaction with the enzyme active site demonstrated promising antimalarial activity and deserve further optimization and investigations.

**Table 2:** Antimalarial activity test for the synthesized compounds at a dose of 48.46 µmol/kg*

Test compounds	Structure	Dose (mg/kg)	% Parasitemia	% Suppression	Mean survival time (day)
9		22.33	34.7 ± 4.1	33.3	8.6 ± 1.8
10		26.67	33.0 ± 6.7	36.5	6.8 ± 0.7
11		19.19	30.0 ± 6.7	42.3	8.7 ± 0.7
12		25.68	67.9 ± 3.7	− 30.5	7.8 ± 1.2
13		23.55	24.0 ± 1.7	53.9	9.5 ± 1.9
14		19.89	15.5 ± 4.1	70.2	9.0 ± 2.0
15		38.18	5.0 ± 1.4	90.4	7.7 ± 1.4
NC**	–	1 mL/100 g	52.0 ± 2.8	0.0	7.0 ± 1.2
CQ	–	25	0	100	ND

^*^The equimolar concentration of the synthesized compounds as compared to the reference drug, CQ. **Values are mean ± standard deviations, p < 0.05. NC, negative control; CQ, chloroquine phosphate; ND, no mouse death was recorded during the study period

#### Molecular docking study

A molecular simulation study was performed to justify the potent in vitro antipromastigote activity of compound **13**, which has desirable fitting pattern in the LmPTR1 pocket (active site) characterized by lower binding free energy (− 9.8 kcal/mol). Inspection of the fitting pattern of compound **13** (Fig. 3) illustrated H-bonding interactions with both Arg287 and Tyr283 through its diethylaminomethylsulphonamide fragment. Additionally, strong hydrophobic interactions with His241 of the catalytic residue were observed. Furthermore, the 1-phenyl-3-tolylpyrazole scaffold of compound **13** substantially anchored within the side chains of Tyr283, Phe113, Gln186, Leu188, Leu226, and Val230 in the Lm-PTR1 pocket. The subsequent hydrophobic interactions were implicated in the desirable in vitro antipromastigote activity of different compounds. The finding was in line with prior reports on Lm-PTR1 [32, 33]. More importantly, the phenylhydrazonomethylene moiety displayed H-bonding interactions with co-factor NADPH and Tyr194 (three H-bonds). It also has strong π-π stacking modes with NADPH and Phe113. The aforementioned desirable interactions of hydrophilic and hydrophobic nature would firmly positioned compound **13** in the Lm-PTR1 pocket resulting in deactivation of PTR1 enzyme. Generally speaking, the in vitro antipromastigote activity and subsequent molecular docking results necessitated additional rigorous experimental protocols to confirm the broad-spectrum antipromastigote activity of compound **13** against various isolates of *Leishmania* parasite and different animal models.

#### In vitro cytotoxicity results

The in vitro cytotoxicity of compound **13** was assessed against VERO cells at different concentrations. The result revealed that 117.79 µM of compound **13** was required to suppress the growth and proliferation of 50% of cells (CC_50_) which is 194-fold higher than the amount required to kill 50% of *Leishmania aethiopica* isolates (Table 3) reaffirming its selectivity [34]. Moreover, it exhibited marked selectivity and pronounced safety profile compared to the conventional drug miltefosine.

**Table 3:** CC_50_ value of compound **13** against normal VERO cells and its selectivity index

Test compounds	Structure	CC_50_ (µM)	IC_50_ (µM)	SI
13		117.79	0.60442	194.88
Miltefosine	–	99.7	7.897	12.63

CC_50_ is the concentration at which 50% of the VERO cells survive and IC_50_ of antileishmanial activity. SI is the selectivity index, SI = CC_50_/IC_50_

#### Acute toxicity results

The in vivo acute toxicity study of compound **13** in mice model revealed that it was devoid of any death at all dose (50, 100, 200, and 300 mg/kg) in the study period (14 days). In addition, physical and gross behavioral observations of the mice also suggested no visible symptoms of toxicity even at the highest dose of 300 mg/kg. Moreover, compound **13** was devoid of any inherent dose-dependent toxicity or death after intraperitoneal injection (≤ 140 mg/kg) within 24 h or the entire study period.

## Experimental

### Materials

#### Chemicals and reagents

Salicylhydrazide, phenyl hydrazine, 2-(4-bromophenyl)-1-(1-phenylethylidene)-hydrazine, 3-phenyl-1-(4-methylphenyl)-1*H*-pyrazole-4-carboxaldehyde, *N*,*N*-Dimethylaminomethylene-4-[3-(4-methylphenyl)-4-formyl-1*H*-pyrazol-1-yl]-benzenesulfonamide, HCl, ethyl acetate, hydrazine hydrate, acetonitrile, chloroform, ethanol, absolute methanol, AlamarBlue^®^, dimethyl sulfoxide (DMSO), acetic acid, phosphorous oxychloride (donated by Drug Discovery Center, Department of Pharmaceutical Chemistry, Faculty of Pharmacy, Alexandria University, Egypt), distilled water, iodine, Giemsa stain, Tween 80, 1% acacia gum, Roswell Park Memorial Institute 1640 (RPMI-1640) medium, heat-inactivated fetal calf serum (HIFCS) and penicillin–streptomycin solution were used in the study. The aforementioned chemicals and reagents were analytical grade and used without further purification.

#### Instruments and apparatuses

Melting points were determined in open capillaries using electro-thermal 9100 melting point apparatus at the Ethiopian Food and Drug Administration, Addis Ababa, Ethiopia, and were uncorrected. Elemental microanalysis was conducted using a Perkin Elmer 2400 elemental analyzer at the Microanalytical Unit, Faculty of Science, Cairo University, Egypt. The FTIR spectra in nujol were recorded with the SHIMADZU 8400SP FT-IR spectrophotometer (Shimadzu Corporation, Nakagyo-Ku, Kyoto, Japan) in the range of 4000 to 500 cm^−1^ at Ethiopian Pharmaceutical Manufacturing (EPHARM), Addis Ababa, Ethiopia, and nuclear magnetic resonance (NMR) spectral data were performed on Bruker Avance DMX400 FT-NMR spectrometer (Bruker, Billerica, MA, USA) using tetramethylsilane (TMS) as an internal standard in Department of Chemistry, Faculty of Science, AAU, Ethiopia. BIO-PLUS microscope was used to count parasites at the Department of Pharmaceutical Chemistry and Pharmacognosy, School of Pharmacy, AAU, Ethiopia. Enzyme-linked Immunosorbent assay (ELISA) plate was also used to determine the absorbance for samples in the antipromastigote assay at Ethiopian Health and Nutrition Research Institute, Addis Ababa, Ethiopia.

#### Experimental animals and strains

Swiss albino mice of either sex weighing 24–30 *g* and 6-week-old donated by the Ethiopian Health and Nutrition Institute, Addis Ababa, Ethiopia were used in the antimalarial activity and acute toxicity study of the target compounds. The mice were housed in cages, maintained in a standard pelleted diet, and acclimatized to the laboratory conditions (temperature of 23–25 ℃, relative humidity of 60–65, and a light/dark cycle of 12 h) for 7 days before each experiment. The rodent malaria parasite, *Plasmodium berghei* ANKA strain was obtained from Biomedical Laboratory at the Department of Biology, Faculty of Science, AAU, Ethiopia. The *Leishmania aethiopica* isolates used in the study were acquired from the Leishmania Diagnostic and Research Laboratory (LDRL), School of Medicine, AAU, Ethiopia.

#### Culture medium and conditions

A complete culture medium was prepared from RPMI-1640, 10% HIFCS, 1% penicillin–streptomycin, and 1% L-glutamine for the in vitro antipromastigote assay. The *Leishmania aethiopica* isolate was grown first on Novy-MacNeal-Nicolle (NNN) medium and then in tissue-culture flasks containing RPMI-1640 medium supplemented with 10% HIFCS and 1% 100 IU penicillin/mL-100 µg/mL streptomycin solution at 22 ℃ [35].

#### Reference drugs

Chloroquine phosphate (Ethiopian Pharmaceutical Manufacturing (EPHARM), Addis Ababa, Ethiopia) was used as a reference drug in the determination of the antimalarial activities of the target compounds. Amphotericin B deoxycholate (Fungizone^®^, ER Squibb, Middlesex, UK) and miltefosine/hexadecylphosphocholine (AG Scientific, San Diego, CA, USA) were employed as reference drugs in the in vitro antileishmanial activity testing of the synthesized compounds.

## Methods

### Synthesis of target compounds

**Fig. 2:**
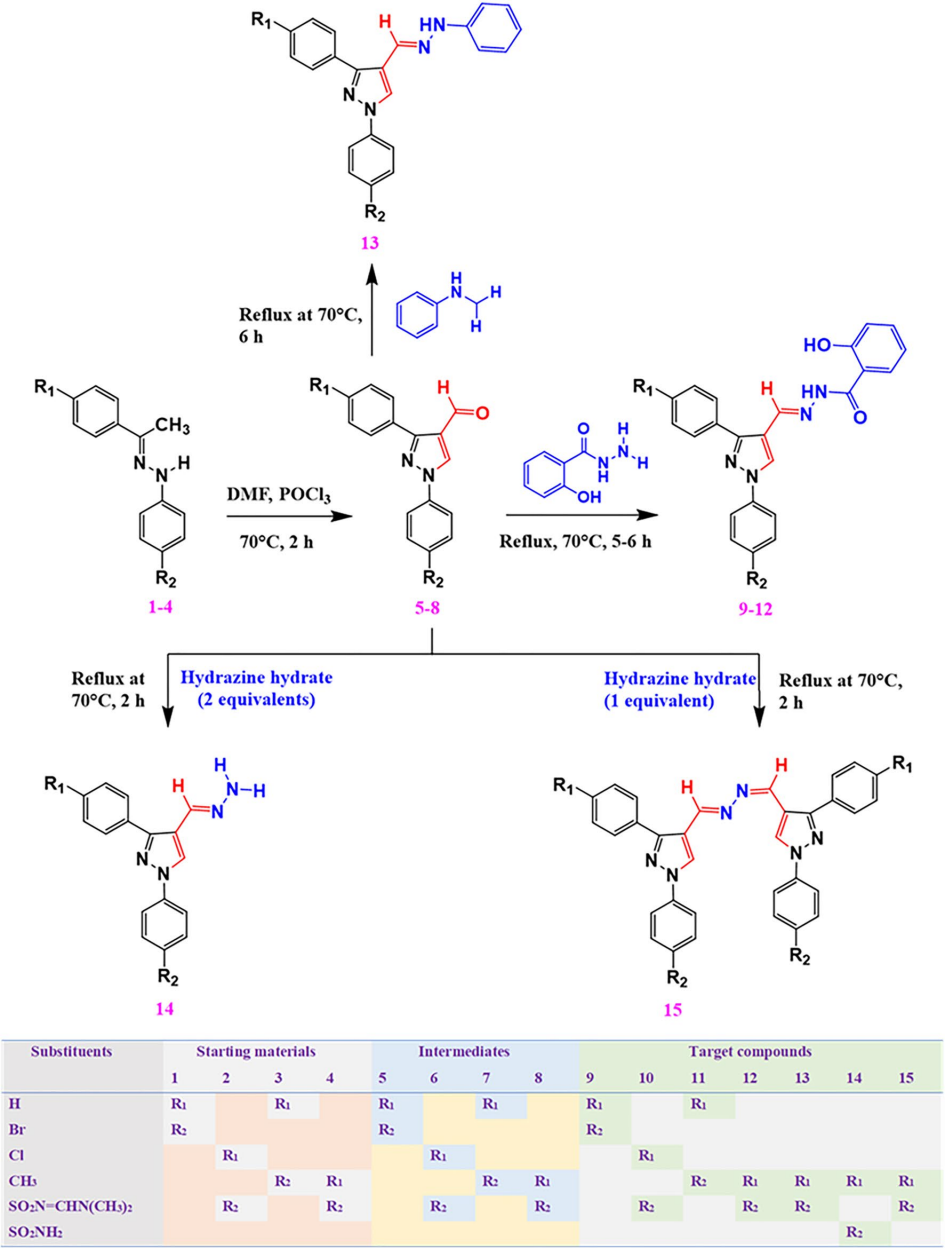
Schematic representation of the different reaction routes used in the hydrazine-coupled pyrazole derivatives

Synthesis of the target compounds **(9–15)** was realized through the condensation of selected pyrazole aldehydes with NH_2_-bearing nucleophiles such as salicyl hydrazide, phenylhydrazine, and hydrazine hydrate [27] as depicted in Fig. 2. Details for the general reaction conditions and purification techniques employed in the synthesis of the target compounds are summarized below.

***General procedure for the synthesis of reaction intermediates (4–8) °***

To an ice-cold DMF (6.93 mL, 89.5 mmol), 2.42 *g* (15.8 mmol) phosphorous oxychloride was added dropwise with continuous stirring for 30 min. The mixture was further stirred for 45 min. Accordingly, the compounds (**1–4**) (2.25 *g*, 6.88 mmol) were added to the mixture and heated at 70 ℃ for 2 h. After cooling, each solution was poured into crushed ice (55 *g*) in water (100 mL). Finally, the corresponding mixture was boiled, cooled and the resulting precipitate was filtered, dried, and recrystallized from methanol to obtain pure products (**5–8**) [27]. As a representative of the synthesized reaction intermediates, the ^1^H NMR data of compound **4** is presented as follows.

***1-(bromophenyl)-3- phenyl-1H-pyrazole-4-carboxaldehyde (4)***

IR (Nujol) (cm^−1^): 1672 (C = O) and 1598 (C = N). ^1^H NMR (CDCl_3_/CCl_4_) ppm: 7.41–7.45 (*t*, 1H, phenyl-C_4_ H), 7.53–7.56 (*t*, 2H, phenyl-C_3,5_ H), 7.65 (*d*, 2H, J = 8.4 Hz, *p*-bromophenyl-C_3,5_ H), 7.78–7.82 (*m*, 4H, *p*-bromophenyl-C_2,6_ H & phenyl-C_2,6_ H), 8.59 (*s*, 1H, pyrazol-C_5_ H), and 10.10 (*s*, 1H, CHO) (Additional file 1: Figure S1). **Anal. calcd**. for C_16_H_11_BrN_2_O: C, 58.74; H, 3.39; N, 8.56; O, 4.89; Br, 24.42. **Found**: C, 58.74; H, 3.39; N, 8.56; O, 4.89; Br, 24.42.

***General procedure for the synthesis of the target compounds (9–15)***

A mixture of pyrazole aldehyde (**5–8**) and stoichiometric amounts of NH_2_-containing nucleophiles (salicyl hydrazide, hydrazine hydrate, or phenyl hydrazine) were dissolved in ethanol (20 mL) and subsequently acidified with two drops of HCl. The solution was heated under reflux at 70 ℃ for 4 to 6 h with continuous stirring. After cooling, the resulting white, the light-yellow, or yellow precipitate was filtered, washed with a large volume of ethanol, dried, and recrystallized from either ethanol, acetonitrile, methanol/ethyl acetate (v/v 2:1), or acetonitrile/ chloroform (v/v 3:1) to get well-defined crystals of target compounds (**9–15**) [27].

***Nʹ-[(1-(4-bromophenyl)-3-phenyl-1H-pyrazol-4-yl) methylene] salicylhydrazide (9)***

IR (Nujol) (cm^−1^): 3334 (OH), 3242 (NH), 1642, (C = O), 1611 and 1598 (C = N). ^1^H NMR (CDCl_3_/DMSO-d_6_) ppm: 6.79–6.85 (*t*, 1H, phenyl-C_4_ H), 6.90 (*d*, 1H, J = 8.3 Hz, hydroxyphenyl-C_4_ H), 7.27–7.49 (*m*, 4H, phenyl-C_3,5_ H & C_2,6_ H), 7.57–7.67 (*2d*, 2H each, J = 8.5 Hz each, *p*-bromophenyl-C_3,5_ H & C_2,6_ H), 7.85 (*d*, 3H, J = 7.8 Hz, hydroxyphenyl-C_3,5_ H & C_6_ H), 8.56 (*s*, 1H, pyrazol-C_5_ H), 8.76 (*s*, 1H, CH = N), 11.67 (*s*, 1H, NH), and 12.2 (*s*, 1H, OH) (Additional file 2: Figure S2). **Anal. calcd**. for C_23_H_17_BrN_4_O_2_: C, 59.88; H, 5.39; N, 12.15; O, 6.94; Br, 17.32. **Found**: C, 59.88; H, 5.39; N, 12.15; O, 6.94; Br, 17.32. **Yield**: 84.17%.

***Nʹ-[(3-(4-chlorophenyl)-1-((4-N,N-dimethylaminomethylenesulfonamido)phenyl)-1H-pyrazol-4-yl) methylene] salicylhydrazide (10)***

IR (Nujol) (cm^−1^): 3296 (NH), 1691 (C = O), 1620, 1593 (C = N), 1339 and 1148 (SO_2_). ^1^H NMR (DMSO-d_6_) ppm: 2.98, 3.15 (*2 s*, 2CH_3_, N(CH_3_)_2_), 6.98 (*m*, 2H, hydroxyphenyl-C_3,5_ H), 7.4- 7.5 (*t*, 1H, hydroxyphenyl-C_4_ H), 7.65 (*d*, 2H, J = 8.4 Hz, *p*-chlorophenyl-C_3,5_ H), 7.8–7.9 (*2d*, 2H, J = 8.4 Hz, *p*-chlorophenyl-C_2,6_ H & J = 7.9 Hz, hydroxyphenyl-C_6_ H), 7.92 (*d*, 2H, J = 8.7 Hz, benzenesulfonamide-C_3,5_ H), 8.20 (*d*, 2H, J = 8.7 Hz, benzenesulfonamide-C_2,6_ H), 8.28 (*s*, 1H, pyrazol-C_5_ H), 8.57 (*s*, 1H, SO_2_N = CH), 9.16 (*s*, 1H, CH = N), 11.83 (*s*, 1H, NH), and 12.00 (*s*, 1H, OH) (Additional file 3: Figure S3). **Anal. calcd**. for C_26_H_23_ClN_6_O_4_S: C, 55.92; H, 3.94; N, 15.65; O, 11.92; S, 5.97; Cl, 6.60. **Found**: C, 55.92; H, 3.94; N, 15.65; O, 11.92; S, 5.97; Cl, 6.60. **Yield**: 66.15%.

***Nʹ-[(3-phenyl-1-(4-methylphenyl)-1H-pyrazol-4-yl)methylene] salicylhydrazide (11)***

IR (Nujol) (cm^−1^): 3262 (OH), 3150 (NH), 1634 (C = O), 1615 and 1598 (C = N). ^1^H NMR (CDCl_3_/CCl_4_) ppm: 2.4 (*s*, 3H, *p*-tolyl-CH_3_), 6.80–6.86 (*t*, 1H, hydroxyphenyl-C_4_ H), 7.0 (*d*, 1H, J = 8.3 Hz, phenyl-C_4_ H), 7.26–7.35 (*m*, 4H, phenyl-C_3,5_ H & C_2,6_ H), 7.53–7.67 (*2d*, 2H each, J = 8.5 Hz each, *p*-tolyl-C_3,5_ H & C_2,6_ H), 7.83 (*d*, 3H, J = 7.8, hydroxyphenyl-C_3,5_ H & C_6_ H), 8.54 (*s*, 1H, pyrazol-C_5_ H), 8.72 (*s*, 1H, CH = N), 11.61 (*s*, 1H, NH), and 12.00 (*s*, 1H, OH) (Additional file 4: Figure S4). **Anal. calcd**. for C_24_H_20_N_4_O_2_: C, 72.71; H, 5.08; N, 14.13; O, 8.07. **Found**: C, 72.71; H, 5.08; N, 14.13; O, 8.07. **Yield**: 83.03%.

***Nʹ-[(1-((4-(N,N-dimethylaminomethylenesulfonamido)-phenyl)-3-(4-methylphenyl)-1H-pyrazol-4-yl) methylene]-salicylhydrazide (12)***

IR (Nujol) (cm^−1^): 3292 (NH), 1635 (C = O), 1622 and 1593 (C = N), 1345 and 1148 (SO_2_). ^1^H NMR (DMSO-d_6_) ppm: 2.4 (*s*, 3H, *p*-tolyl-CH_3_), 2.95,3.19 (2* s*, 2CH_3_, N(CH_3_)_2_), 6.9–7.0 (*m*, 2H, hydroxyphenyl-C_3,5_ H), 7.37 (*d*, 2H, J = 7.9 Hz, *p*-tolyl-C_3,5_ H), 7.43–7.48 (*t*, 1H, hydroxyphenyl-C_4_ H), 7.66 (*d*, 2H, J = 7.9 Hz, *p*-tolyl-C_2,6_ H), 7.86 (*d*, 1H, J = 7.6 Hz, hydroxyphenyl-C_6_ H), 7.92 (*d*, 2H, J = 8.7 Hz, benzenesulfonamide-C_3,5_ H),8.20 (*d*, 2H, J = 8.7 Hz, benzenesulfonamide-C_2,6_ H), 8.28 (*s*, 1H, pyrazol-C_5_ H), 8.57 (*s*, 1H, SO_2_N = CH), 9.14 (*s*, 1H, CH = N), 11.85 (*s*, 1H, NH), and 12.0 (*s*, 1H, OH) (Additional file 5: Figure S5). **Anal. calcd**. for C_27_H_26_N_6_O_4_S: C, 61.12, H, 4.94; N, 15.84; O, 12.06; S, 6.04. **Found**: C, 60.45, H, 3.71; N, 16.27; O, 12.39; S, 6.03. **Yield**: 95.5%.

***N,N-dimethylaminomethylene-4-[(3-(4-methylphenyl)-4-(phenylhydrazonomethylene))-1H-pyrazol-1-yl]benzenesulfonamide (13)***

IR (Nujol) (cm^−1^): 3292 (NH), 1615 and 1593 (C = N), 1340 and 1148 (SO_2_). ^1^H NMR (DMSO-d_6_) ppm: 2.40 (*s*, 3H, *p*-tolyl-CH_3_), 2.95,3.19 (2* s*, 2CH_3_, N(CH_3_)_2_), 6.76 (*t*, 1H, phenyl-C_4_ H), 7.03 (*d*, 2H, J = 7.8 Hz, phenyl-C_3,5_ H), 7.21 (*d*, 2H, J = 7.8 Hz, phenyl-C_2,6_ H), 7.36 (*d*, 2H, J = 8.0 Hz, *p*-tolyl-C_3,5_ H), 7.65 (*d*, 2H, J = 8.0 Hz, *p*-tolyl-C_2,6_ H), 7.89–7.97 (*d*, 2H, J = 8.8 Hz, benzenesulfonamide-C_3,5_ H & pyrazol-C_5_ H), 8.15 (*d*, 2H, J = 8.8 Hz, benzenesulfonamid-C_2,6_ H), 8.27 (*s*, 1H, SO_2_N = CH), 8.99 (*s*, 1H, CH = N), and 10.25 (*s*, 1H, NH) (Additional file 6: Figure S6). **Anal. calcd**. for C_26_H_26_N_6_O_2_S: C, 64.18; H, 5.38; N, 17.27; O, 6.58; S, 6.59. **Found**: C, 64.18; H, 4.68; N, 17.27; O, 6.59; S, 6.59. **Yield**: 61.64%.

***4-[3-(4-methylphenyl)-4-hydrazonomethylene-1H-pyrazol-1-yl] benzenesulfonamide (14)***

IR (Nujol, cm^−1^): 3350 and 3234 (NH_2_), 1625 and 1596 (C = N), 1340 and 1154 (SO_2_). ^1^H NMR (DMSO-d_6_): 2.4 (*s*, 3H, *p*-tolyl CH_3_), 7.38 (*d*, 2H, J = 8.0 Hz, *p*-tolyl C_3,5_ H), 7.49 (*s*, 2H, CH = N-NH_2_), 7.65 (*d*, 2H, J = 8.0 Hz, *p*-tolyl C_2,6_ H), 7.97, 8.23 (*2d*, 4H, J = 8.7 Hz, benzenesulfonamide-C_3,5_ H & C_2,6_ H), 8.69 (*s*, 1H, pyrazole-C_5_ H), and 9.27 (*s*, 1H, CH = N) (Additional file 7: Figure S7). **Anal. calcd**. for C_20_H_22_N_6_O_2_S: C, 58.52; H, 5.40; N, 20.47; O, 7.79; S, 7.81. **Found**: C, 57.45; H, 4.82; N, 19.7; O, 9.0; S, 9.02. **Yield**: 75.8%.

***Bis-[N,N-dimethylaminomethylene-4-(3-(4-methylphenyl)-4-(hydrazonomethylene)-1H-pyrazole-1-yl) benzenesulfonamide] (15)***

IR (Nujol) (cm^−1^): 1626 (C = N), 1340 and 1152 (SO_2_). ^1^H NMR (DMSO-d_6_): 2.4 (*s*, 3H, *p*-tolyl CH_3_), 2.95, 3.2 (*2 s*, 2CH_3_, N(CH_3_)_2_), 7.36, 7.64 (*2d*, 2H each, J = 8.0 Hz each, *p*-tolyl C_3,5_ H & C_2,6_ H), 7.92, 8.17 (*2d*, 2H each, J = 8.7 Hz, benzenesulfonamide-C_3,5_ H & C_2,6_ H), 8.26 (*s*, 1H, pyrazol-C_5_ H), 8.67 (*s*, 1H, SO_2_N = CH), and 9.23 (*s*, 1H, CH = N) (Additional file 8: Figure S8). **Anal. calcd**. for C_40_H_40_N_10_O_4_S_2_: C, 60.90, H, 5.12; N, 17.75; O, 8.11; S, 8.13. **Found**: C, 60.90; H, 5.11; N, 17.75; O, 8.11; S, 8.13. **Yield**: 67.63%.

### Preparation of stock and working solutions

The entire test compound was dissolved in DMSO to a final concentration of 1 mg/mL. The final DMSO concentration was adjusted not to exceed v/v 1% to avoid its inhibitory effect against parasite proliferation or change in morphology. Six different concentrations of test compounds, ranging from 10 µg/mL to 0.04 µg/mL, were prepared by three-fold serial dilutions. Amphotericin B deoxycholate (with a concentration ranging from 0.5 µg/mL to 0.002 µg/mL) and miltefosine (with a concentration ranging from 40 µg/mL to 0.16 µg/mL) were used as a positive control and DMSO (v/v 1%) in complete media was used as a negative control to compare the antileishmanial activities of test compounds [36]. All the prepared drugs were stored at − 20 ℃ until used in the experiment.

### In vivo* a*ntimalarial activity test

The in vivo antimalarial activity of the synthesized compounds was determined using the four-day standard suppressive test [37] using Swiss albino mice donated by the Ethiopian Health and Nutrition Institute, Addis Ababa, Ethiopia. Briefly, Swiss albino mice were infected with *Plasmodium berghei* ANKA strain (0.2 mL of 2 × 10^7^ parasitized erythrocytes) through intraperitoneal injection. After 2 h of infection, the mice were weighed and randomly divided into nine groups of five mice per cage. Groups **1–7** received each test compound (48.4 µg/mL) dissolved in a vehicle containing 7% Tween 80, 3% ethanol, and water. Group **8** was treated with chloroquine phosphate (25 mg/kg) suspended in 7% Tween 80, 3% ethanol, and water which served as a positive control. Group **9** received 2 mL/100 *g* of the negative control (vehicle 7% Tween 80, 3% ethanol, and water). On day four (96 h post-infection), a blood smear was taken from the tail of each mouse, fixed with absolute ethanol, and stained with Giemsa stain. Then, the level of parasitemia was determined microscopically by counting four fields of approximately 100 erythrocytes per field. Finally, the antimalarial effects of control groups and test compounds were expressed in terms of blood parasitemia, percent suppression and mean survival time of mice [38].

### In vitro antileishmanial activity test

The antileishmanial activity of the test compounds was evaluated in vitro against isolates of *Leishmania aethiopica* promastigotes. 100 µL of the parasite suspension containing 3 × 10^6^ promastigotes per milliliter was added to 96-well plates. The six different concentrations of test compounds and reference drugs were then added in triplicate. DMSO in complete RPMI media was used as a negative control to compare the antileishmanial activities of the test compounds. After 24 h incubation, 20 µL of fluorochrome AlamarBlue^®^ (12.5 mg resazurin dissolved in 100 mL of distilled water) was added to each well. Then, the absorbance of each well was measured after 4 h of incubation at wavelengths of 492 and 630 nm using an ELISA reader for the quantitative determination of viable cells. Finally, the IC_50_ values were computed from sigmoidal dose–response curves using GraphPad Prism 5.0 software (GraphPad Software, Inc., San Diego, CA, USA) [35].

### Molecular docking study

**Fig. 3:**
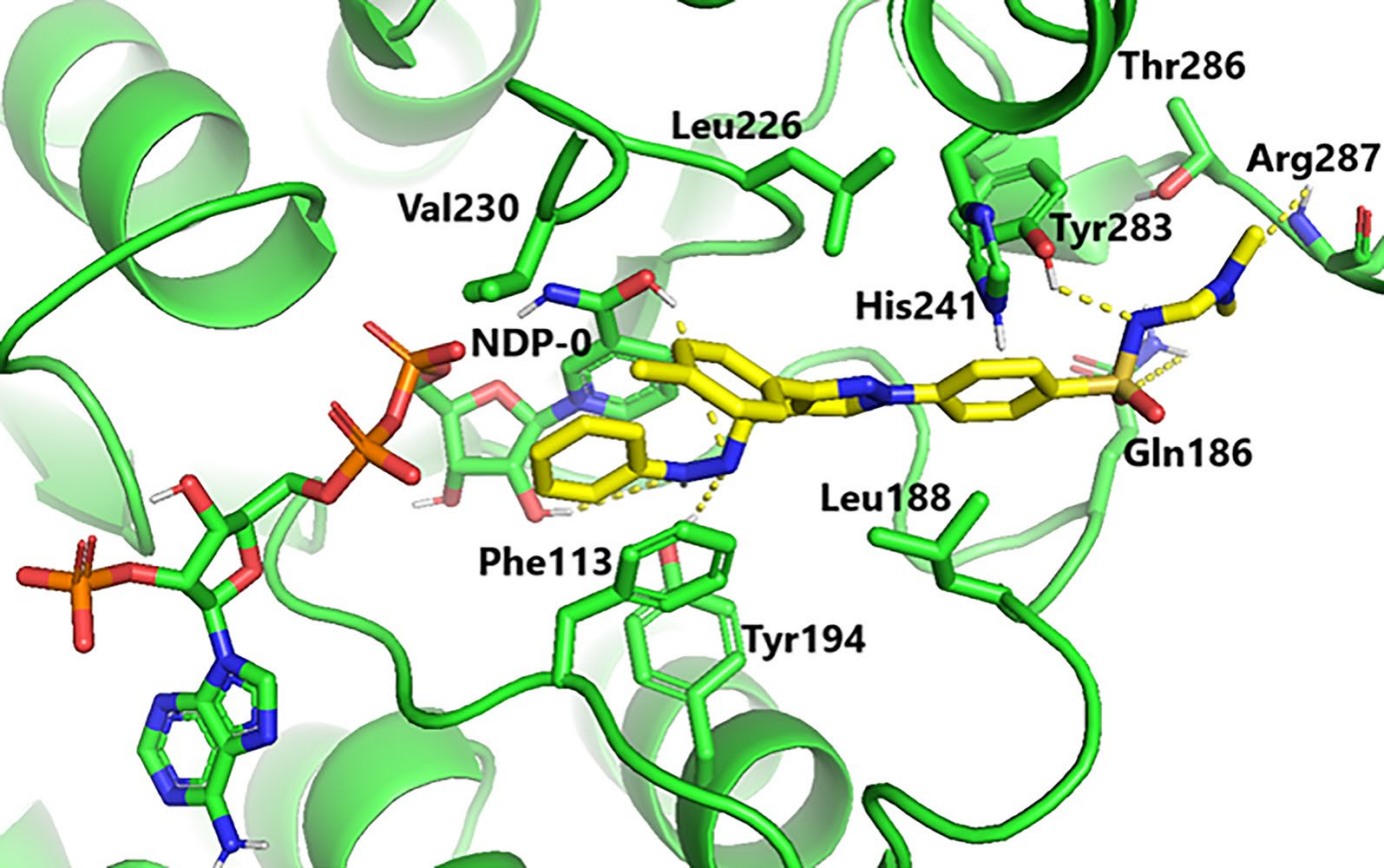
The docking pose of compound **13** as yellow sticks in the binding site of Lm-PTR1(PDB code:2bfm). NDP-0 is referred to as NADPH co-factor

AutoDock Vina was used to perform the molecular simulation study [36] for compound **13** which elicited pronounced antileishmanial activity against *Leishmania aethiopica* promastigotes. The 3D structure of Lm-PTR1 complexed with Trimethoprim was obtained from the Protein Data Bank (PDB ID:2bfm), forming by chain A of the Lm-PTR1 heterodimer employed in the modeling study. Compound **13** was sketched in a manner that minimize energy and the protein was formulated via the Discovery studio suite (V5.1). The Python script (prepare receptor4.py) provided by the MGLTools package (version 1.5.4) was captured to convert protein files to PDBQT format for docking using AutoDock Vina (version 1.1.2). The efficiency of the search algorithm pertained to its default setting. The grid box docking dimensions were − 5.589 Å × 41.846 Å × 68.229 Å, with a spacing of 1 Å to deal with all the possible conformations of the docked molecule. Pymol was used to generate the graphical representations depicted in Fig. 3.

### In vitro cytotoxicity test

The cytotoxicity of compound **13** with promising antileishmanial activity was investigated in vitro against African green monkey kidney cells (VERO cells) [39]. In a 96-well plate, VERO cells (1 × 10^5^ cells per well) were incubated in triplicates with different concentrations of compound **13** (0–100 µM) for 72 h at 37 ℃ incubator with 95% humidity and 5% CO_2_ and the cell viability was determined by measuring the optical density (OD) of formazan. The CC_50_ value (the concentration of compound **13** required to kill 50% of fibroblast cells) was and compared with the standard drug, miltefosine. The CC_50_ and IC_50_ values were computed using the formula: Growth inhibition (%) = $(ODcontrol-ODtest/ODcontrol) \times 100$. In addition, the selectivity indices (SI) were computed using the formula SI = CC_50_/IC_50_. IC_50_ refers to the concentration of compound **13** required to kill 50% of *Leishmania aethiopica*).

### In vivo* a*cute toxicity test

The oral acute toxicity of compound **13** with promising antileishmanial activity was investigated using male Swiss albino mice (~ 20 *g* each). The mice were weighed and divided into five groups of six mice per cage. After fasting them overnight, groups **1–4** received the test compound suspended in 1% acacia gum was administered orally at doses of 50, 100, 200, and 300 mg/kg [37]. Group **5** (served as a control) was treated with the solvent (1% acacia gum) at a maximum dose of 1 mL/100 gm of body weight [40]. In a separate experiment, compound **13** was administered with intraperitoneal injection at a dose of 40, 80, 120, and 140 mg/kg [41]. Then, each mouse was observed for gross acute toxicity signs like sedation, lacrimation, hair erection, blinking, sleep, coma, death, etc. Follow-up continued for 14 days with special attention for the first 24 h.

### Ethical consideration

The protocols that involved experimental animals were assessed and approved by the Institutional Ethics Review Committee, School of Pharmacy, Addis Ababa University. The mice used in the study were donated by Ethiopian Health and Nutrition Institute (EHNI), Addis Ababa, Ethiopia and informed consent was obtained prior to use. Mouse work, such as injection with parasites or extracts, and euthanasia was implemented under general inhalation anesthesia induced with isoflurane (2%) to minimize animal suffering. Mice were euthanized by cervical dislocation at 30 days after parasite infection. In addition, the study finding was reported following the Animal Research Reporting of in vivo Experiments (ARRIVE) guidelines [42] and animals were handled according to the Guide for the Care and Use of Laboratory Animals (https://olaw.nih.gov/sites/default/files/Guide-for-the-Care-and-Use-of-Laboratory-Animals.pdf).

### Statistical analysis

The % suppression and % parasitemia in the antimalarial activity testing were computed using the formulas given below and the resulting data along with the mean survival time was analyzed using Microsoft office excel 2007. The variation in the values of suppressive test was assessed by using one-way analysis of variance (ANOVA) and *p* < 0.05 is considered as stastically significant.

$$\mathrm{Parasitemia }(\mathrm{\%})=\frac{\mathrm{Number \,of \,infected \,red \,blood \,cells}}{\mathrm{Number \,of \,total \,red \,blood \,cells}}\times 100$$

$$\mathrm{Suppression }(\mathrm{\%})=\frac{\mathrm{Parasitemia \,in \,the \,untreated \,group }-\mathrm{Parasitemia \,in \,the \,treated \,group}}{\mathrm{Parasitemia \,in \,untreated \,group}}\times 100$$

## Conclusion

In this study, some hydrazine-coupled pyrazole derivatives were synthesized and screened for their potential antimalarial and antileishmanial activity. Compound **13** displayed promising antileishmanial activity against *Leishmania aethiopica* isolate which is 174- and 2.6-fold more active than miltefosine and amphotericin B deoxycholate, respectively. The docking study also supported the desirable in vitro antileishmanial activity of compound **13**. Moreover, compounds **14** and **15** displayed promising suppression of parasitemia in mice infected with the *Plasmodium berghei* ANKA strain. In general, the hydrazine-coupled pyrazole derivatives demonstrated desirable dual antileishmanial and antimalarial effects, suggesting the importance of the target compounds as potential pharmacophores in the development of safe and efficacious antileishmanial and antimalarial agents.

### Supplementary Information

**Additional file 1: Figure S1.** ^1^H NMR spectrum of compound** 4** in CDCl_3_.

**Additional file 2: Figure S2.** ^1^H NMR spectrum of compound **9** in CDCl_3_.

**Additional file 3: Figure S3.** ^1^H NMR spectrum of compound **10** in DMSO-d_6_.

**Additional file 4: Figure S4.** ^1^H NMR spectrum of compound **11** in CDCl_3_/DMSO-d_6_.

**Additional file 5: Figure S5.** ^1^H NMR spectrum of compound **12** in DMSO-d_6_.

**Additional file 6: Figure S6.** ^1^H NMR spectrum of compound **13** in DMSO-d_6_.

**Additional file 7: Figure S7.** ^1^H NMR spectrum of compound **14** in DMSO-d_6_.

**Additional file 8: Figure S8.** ^1^H NMR spectrum of compound **15** in DMSO-d_6_.

## Data Availability

The datasets that are not included in this article can be shared with the corresponding author upon request.
