# Supplementary figures and images for: Synthesis, antileishmanial, antimalarial evaluation and molecular docking study of some hydrazine-coupled pyrazole derivatives
Source: BMC Chem. 2024 Jan 8;18(1):9. doi: 10.1186/s13065-023-01111-0 (PMC10775556; doi:10.1186/s13065-023-01111-0)

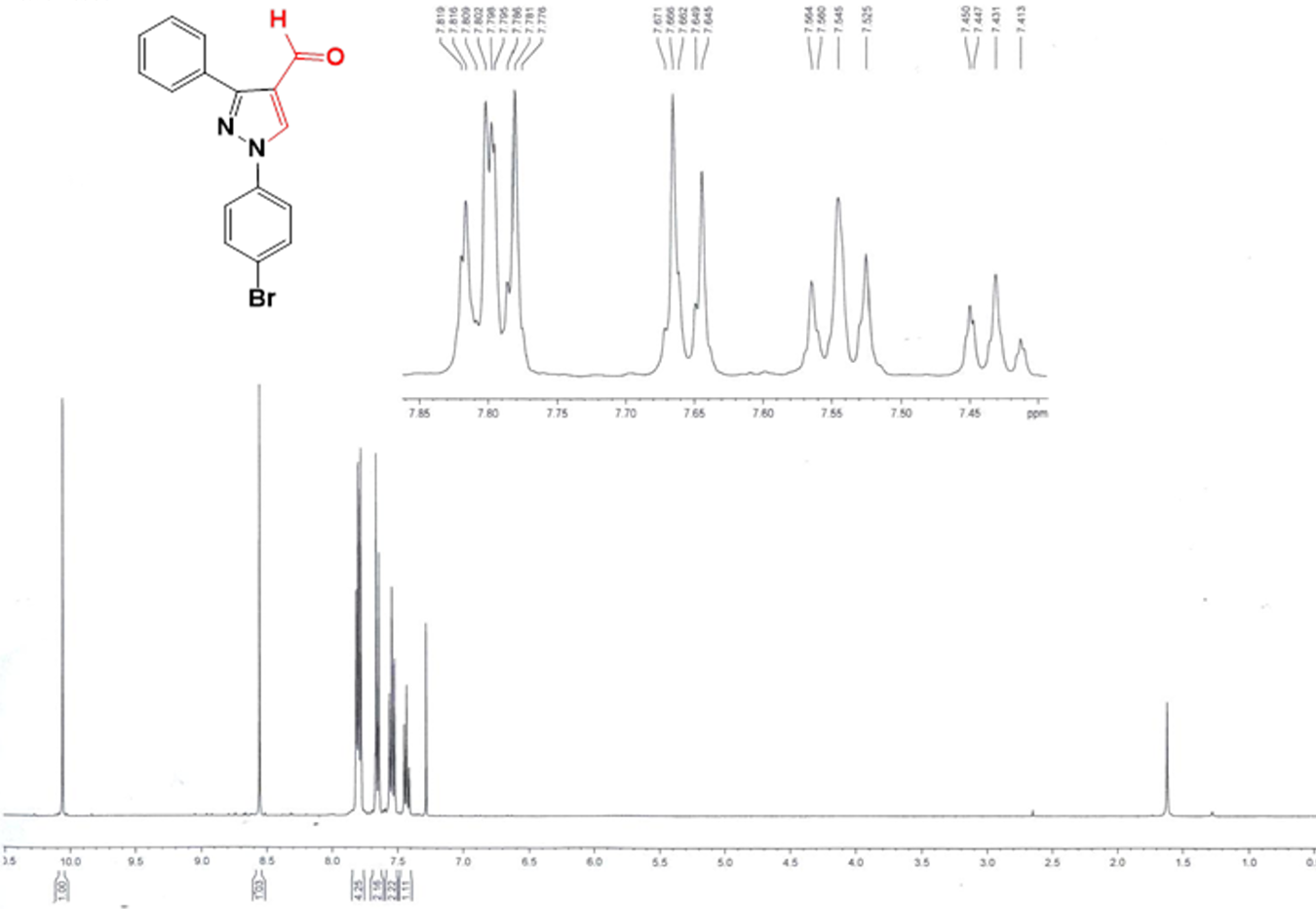


**Figure S1**: 1H NMR spectrum of compound 4 in CDCl3.

Supplement: Supplementary file 1 — Additional file 1: Figure S1. 1H NMR spectrum of compound 4 in CDCl3. [file 13065_2023_1111_MOESM1_ESM.docx]

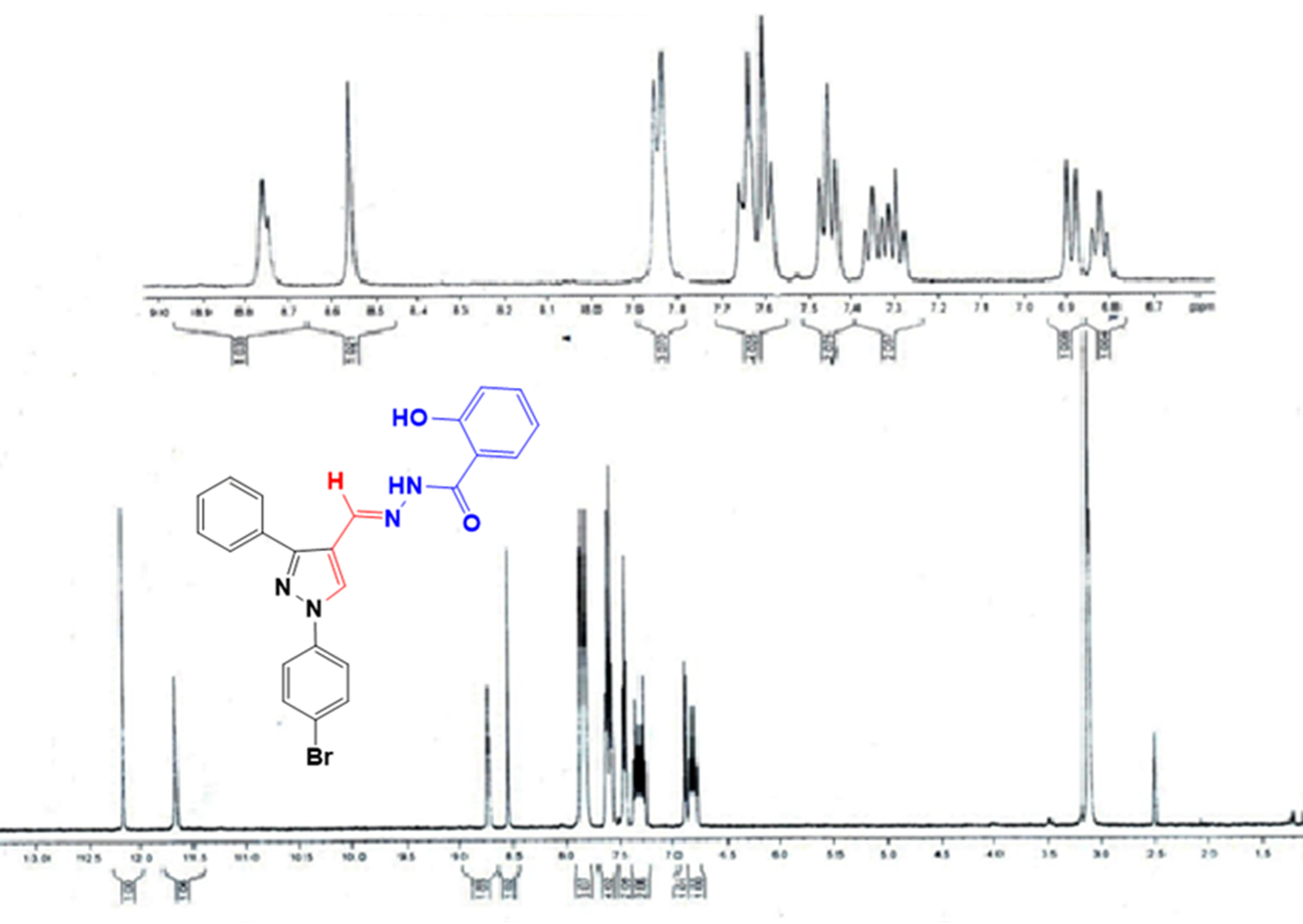


**Figure S2**: 1H NMR spectrum of compound **9** in CDCl3.

Supplement: Supplementary file 2 — Additional file 2: Figure S2. 1H NMR spectrum of compound 9 in CDCl3. [file 13065_2023_1111_MOESM2_ESM.docx]

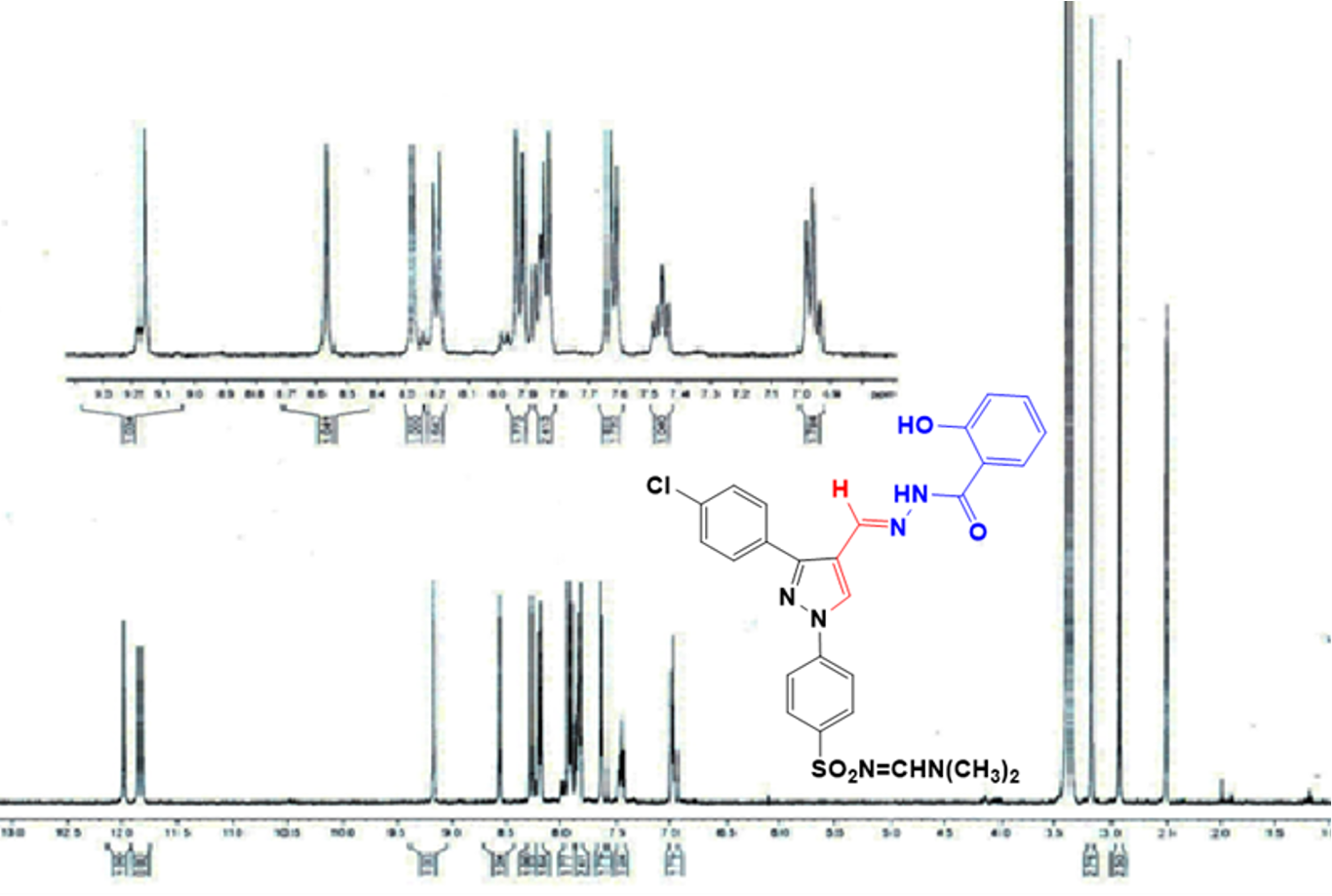


**Figure S3**: 1H NMR spectrum of compound **10** in DMSO-d6.

Supplement: Supplementary file 3 — Additional file 3: Figure S3. 1H NMR spectrum of compound 10 in DMSO-d6. [file 13065_2023_1111_MOESM3_ESM.docx]

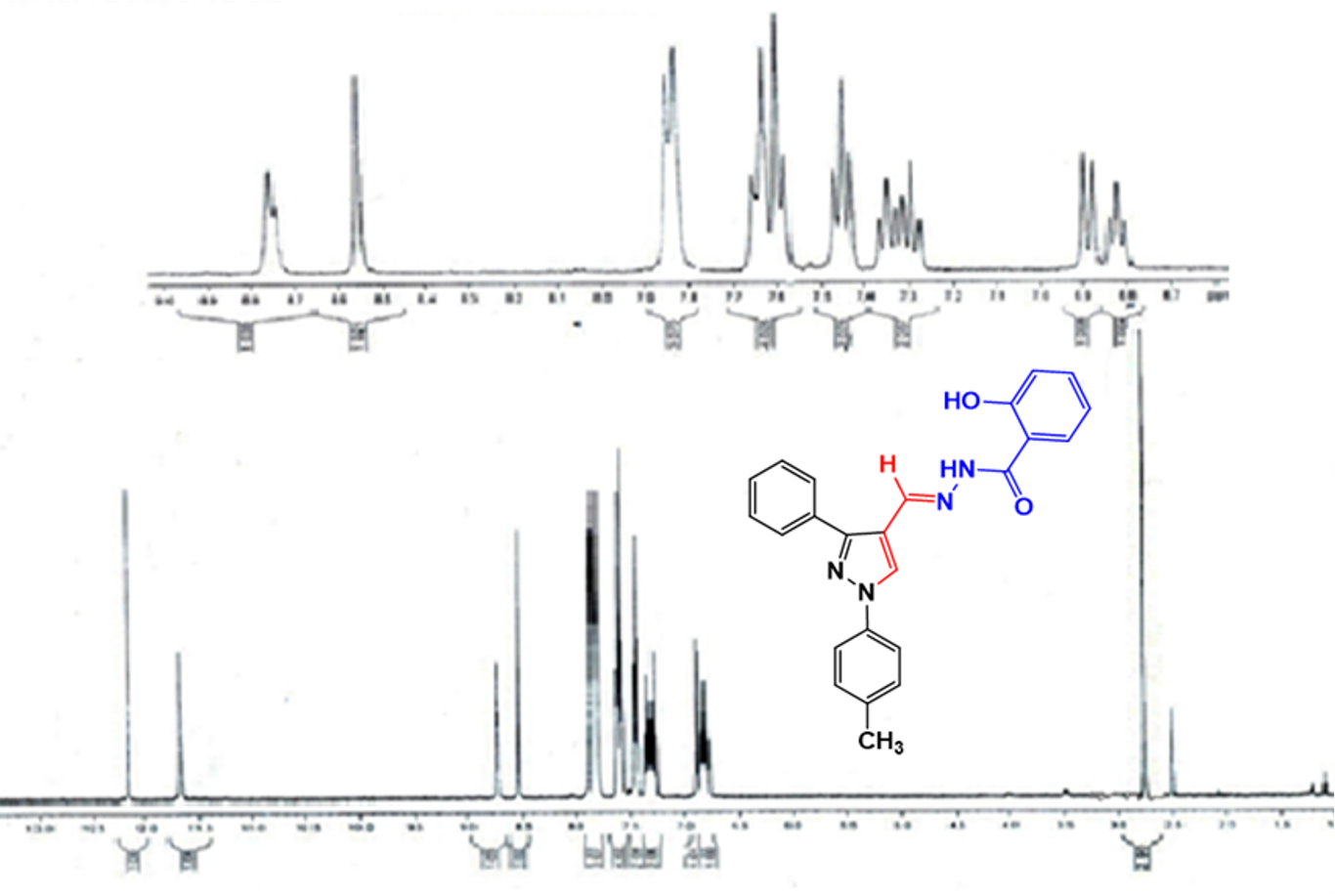


**Figure S4**: 1H NMR spectrum of compound **11** in CDCl3/DMSO-d6.

Supplement: Supplementary file 4 — Additional file 4: Figure S4. 1H NMR spectrum of compound 11 in CDCl3/DMSO-d6. [file 13065_2023_1111_MOESM4_ESM.docx]

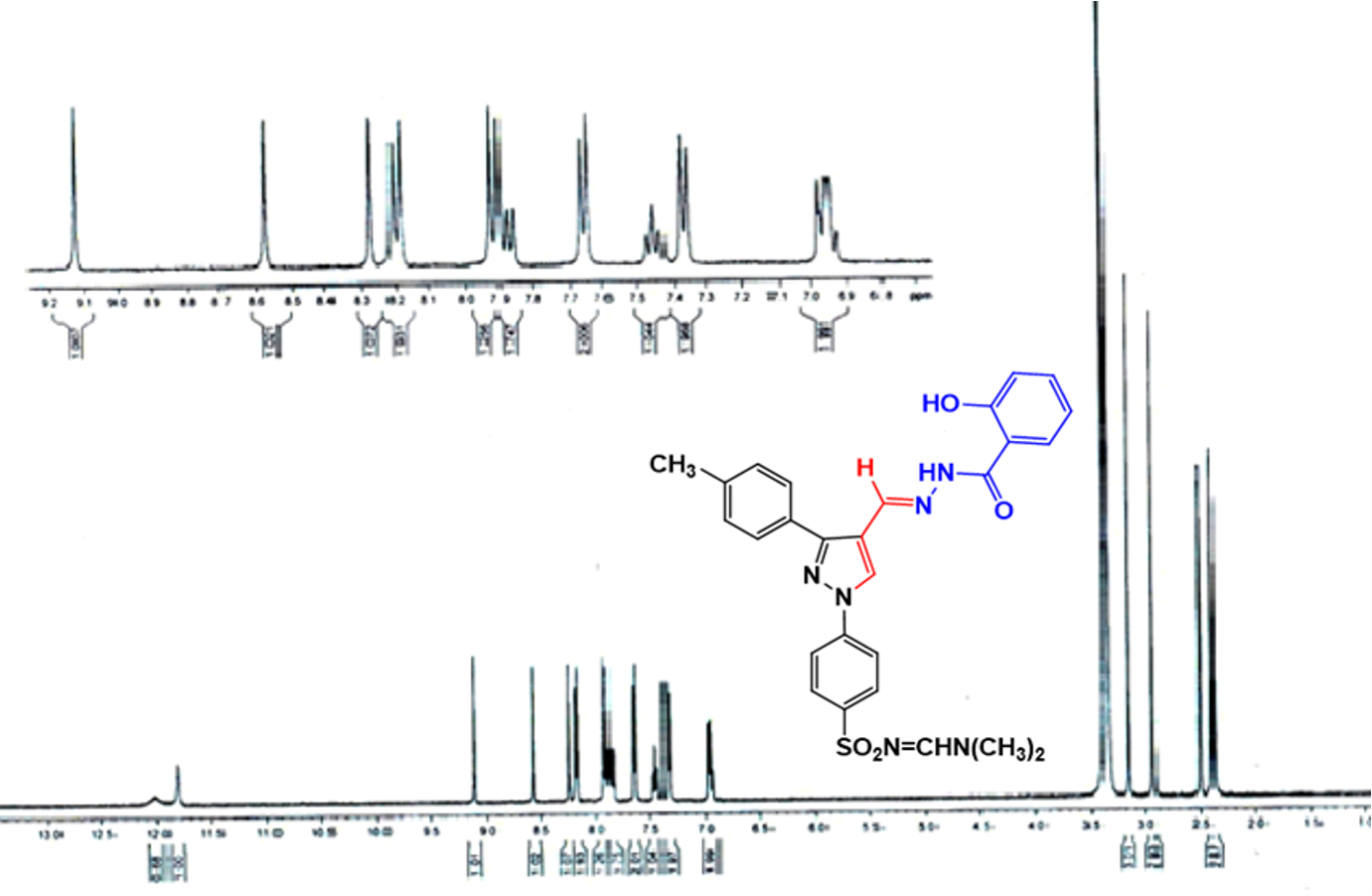


**Figure S5**: 1H NMR spectrum of compound **12** in DMSO-d6.

Supplement: Supplementary file 5 — Additional file 5: Figure S5. 1H NMR spectrum of compound 12 in DMSO-d6. [file 13065_2023_1111_MOESM5_ESM.docx]

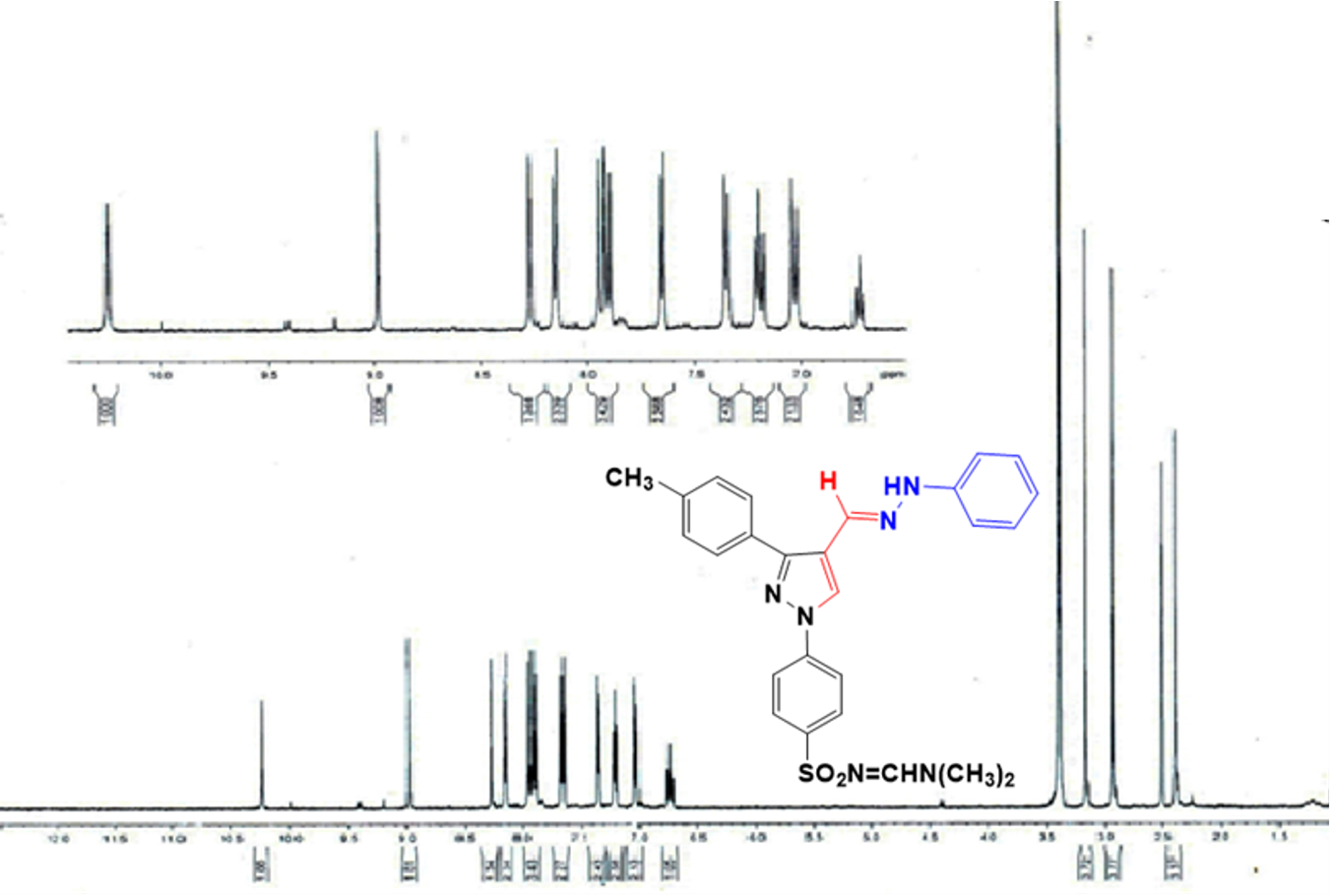


**Figure S6**: 1H NMR spectrum of compound **13** in DMSO-d6.

Supplement: Supplementary file 6 — Additional file 6: Figure S6. 1H NMR spectrum of compound 13 in DMSO-d6. [file 13065_2023_1111_MOESM6_ESM.docx]

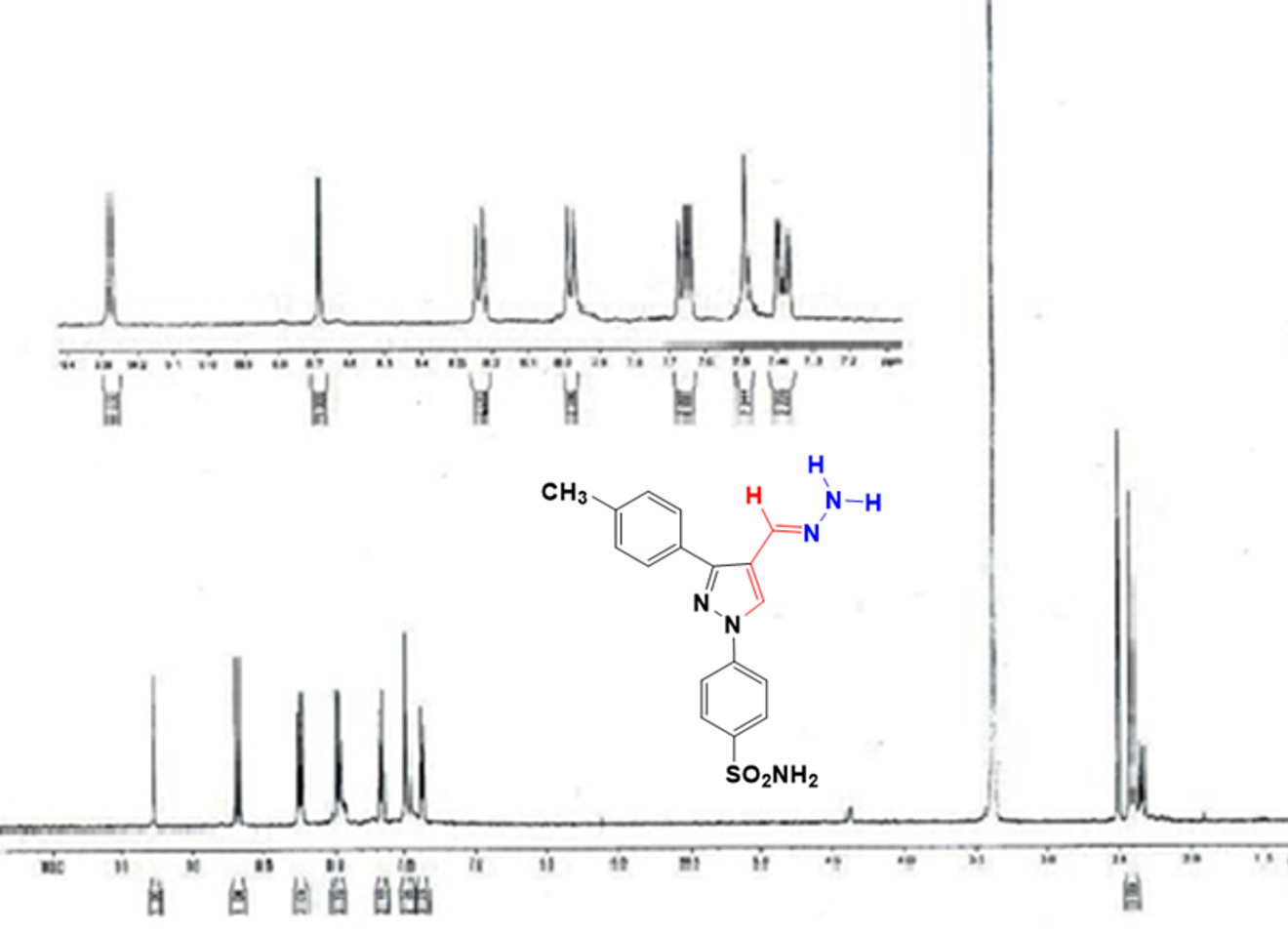


**Figure S7**: 1H NMR spectrum of compound **14** in DMSO-d6.

Supplement: Supplementary file 7 — Additional file 7: Figure S7. 1H NMR spectrum of compound 14 in DMSO-d6. [file 13065_2023_1111_MOESM7_ESM.docx]

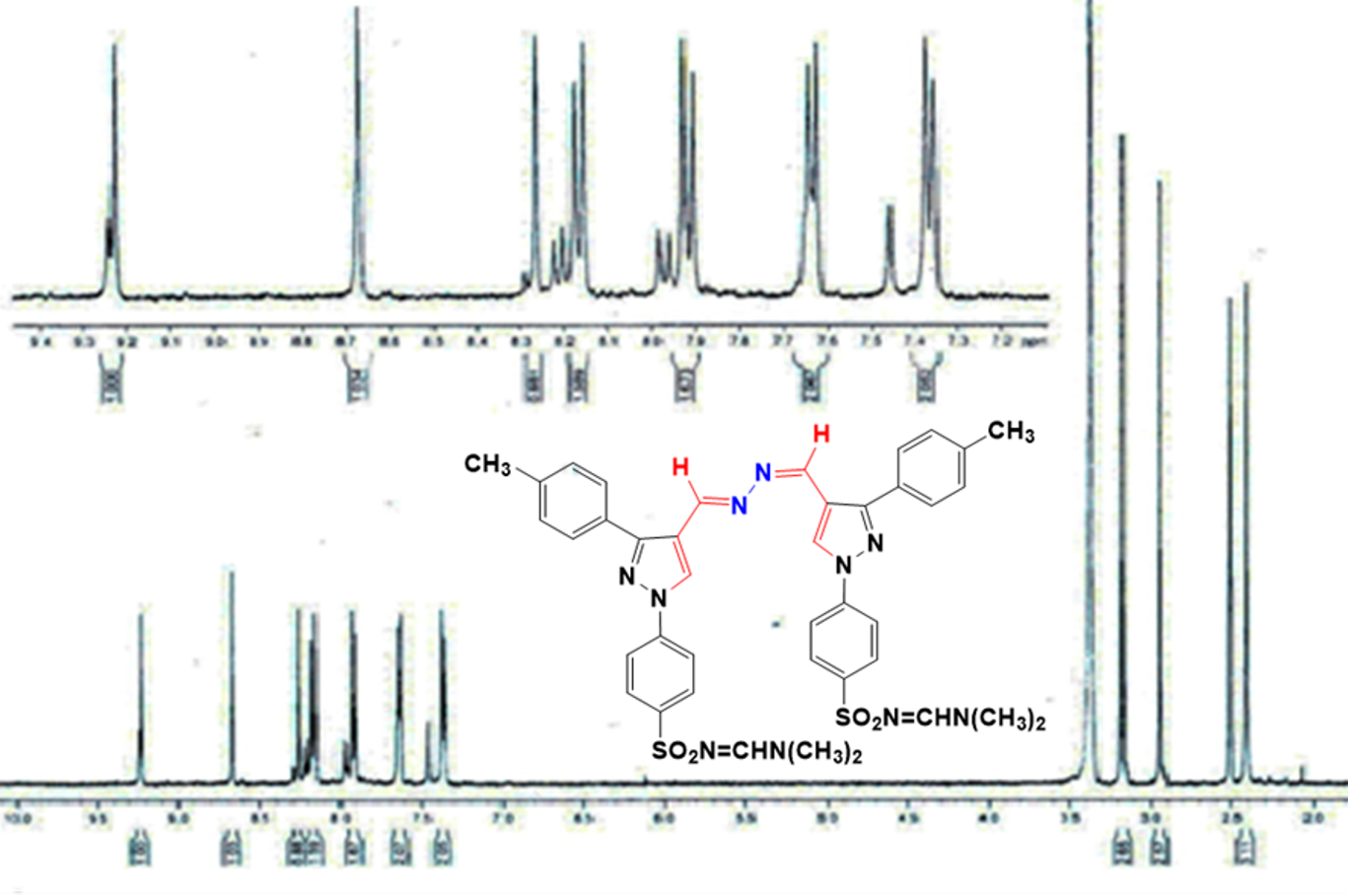


**Figure S8**: 1H NMR spectrum of compound **15** in DMSO-d6.

Supplement: Supplementary file 8 — Additional file 8: Figure S8. 1H NMR spectrum of compound 15 in DMSO-d6. [file 13065_2023_1111_MOESM8_ESM.docx]
